# A multicentre non-blinded randomised controlled trial to assess the impact of regular early specialist symptom control treatment on quality of life in malignant mesothelioma (RESPECT-MESO): study protocol for a randomised controlled trial

**DOI:** 10.1186/1745-6215-15-367

**Published:** 2014-09-19

**Authors:** Samal Gunatilake, Fraser JH Brims, Carole Fogg, Iain Lawrie, Nick Maskell, Karen Forbes, Najib Rahman, Steve Morris, Reuben Ogollah, Stephen Gerry, Mick Peake, Liz Darlison, Anoop J Chauhan

**Affiliations:** RESPECT Meso trial co-ordinator, Portsmouth Hospitals NHS Trust, Portsmouth, England; Consultant Respiratory Physician and Clinical Senior Lecturer, Sir Charles Gairdner Hospital, Perth, Western Australia Australia; Senior Lecturer, University of Portsmouth/Portsmouth Hospitals NHS Trust, Portsmouth, England; Consultant& Honorary Clinical Senior Lecturer in Palliative Medicine, The Pennine Acute Hospitals NHS Trust/The University of Manchester, Manchester, England; Consultant and Reader, North Bristol Lung Centre, Southmead Hospital, Bristol, England; Consultant and Macmillan Professorial Teaching Fellow, Bristol Haematology and Oncology Centre, Bristol, England; Consultant and Senior Lecturer, Oxford Centre for Respiratory Medicine, Director Oxford Respiratory Trials Unit Churchill Hospital, Bristol, England; Healthcare Economics, University College London, London, England; Lecturer, University of Portsmouth, Portsmouth, England; Trial statistician, Centre for Statistics in Medicine, University of Oxford, Oxford, England; Consultant Physician & Senior Lecturer, Department of Respiratory Medicine, Glenfield Hospital, Leicester, England; Nurse Consultant, Mesothelioma UK, Glenfield Hospital, Leicester, England; Consultant Respiratory Physician and Director of Research & Development, Portsmouth Hospitals NHS Trust, Portsmouth, England

**Keywords:** Mesothelioma, Palliative care, Symptom control, Quality of life

## Abstract

**Background:**

Malignant pleural mesothelioma is an incurable cancer caused by exposure to asbestos. The United Kingdom has the highest death rate from mesothelioma in the world and this figure is increasing. Median survival is 8 to 12 months, and most patients have symptoms at diagnosis. The fittest patients may be offered chemotherapy with palliative intent. For patients not fit for systemic anticancer treatment, best supportive care remains the mainstay of management. A study from the United States examining advanced lung cancer showed that early specialist palliative care input improved patient health related quality of life and depression symptoms 12 weeks after diagnosis. While mesothelioma and advanced lung cancer share many symptoms and have a poor prognosis, oncology and palliative care services in the United Kingdom, and many other countries, vary considerably compared to the United States. The aim of this trial is to assess whether regular early symptom control treatment provided by palliative care specialists can improve health related quality of life in patients newly diagnosed with mesothelioma.

**Methods:**

This multicentre study is an non-blinded, randomised controlled, parallel group trial. A total of 174 patients with a new diagnosis of malignant pleural mesothelioma will be minimised with a random element in a 1:1 ratio to receive either 4weekly regular early specialist symptom control care, or standard care. The primary outcome is health related quality of life for patients at 12 weeks. Secondary outcomes include health related quality of life for patients at 24 weeks, carer health related quality of life at 12 and 24 weeks, patient and carer mood at 12 and 24 weeks, overall survival and analysis of healthcare utilisation and cost.

**Discussion:**

Current practice in the United Kingdom is to involve specialist palliative care towards the final weeks or months of a life-limiting illness. This study aims to investigate whether early, regular specialist care input can result in significant health related quality of life gains for patients with mesothelioma and if this change in treatment model is cost-effective. The results will be widely applicable to many institutions and patients both in the United Kingdom and internationally.

**Trial registration:**

Current controlled trials ISRCTN18955704.

Date ISRCTN assigned: 31 January 2014.

**Electronic supplementary material:**

The online version of this article (doi:10.1186/1745-6215-15-367) contains supplementary material, which is available to authorized users.

## Background

Malignant pleural Mesothelioma (MPM) is an incurable cancer of the lining of the lung caused by exposure to asbestos. Prior to controls on asbestos exposure introduced in the 1970s, industrial asbestos exposure was widespread in the United Kingdom (UK), although a much larger workforce involved with lagging and construction continued to be exposed until the early 1980s[1]. Imports of asbestos to the UK only ceased in 1999[2].

The UK now has the highest death rate from MPM in the world[1] and predictions forecast this figure increasing up to 2015 and possibly 2020[3–5]. More precise predictions are hampered by the long lag time between exposure and disease manifestation of at least 30 to 40 years[6]. The projected lifetime risk of MPM for a UK male born in the 1940s is 0.59%, or approximately 1 in 170 of all deaths[3].

There is no cure for mesothelioma. Prognosis is poor, with a median survival of between 8 and 12 months, although long survivors are recognised[7]. A previous study examining health related quality of life (HRQoL) in MPM patients receiving chemotherapy has demonstrated that 92% of patients have 3 or more physical symptoms at presentation[8]. Fatigue (94%), dyspnoea (89%), appetite loss (87%) and pain (85%) were the most common, with fatigue, dyspnoea and pain associated with a worse global HRQoL. Aggressive treatment strategies to prolong life include combinations of surgery, radiotherapy and chemotherapy, but remain controversial with no sound evidence base. At present, the fittest patients with mesothelioma will be offered chemotherapy with palliative intent, which has been shown to improve survival by two to three months in clinical trials[9]. For less fit patients, best supportive care remains the mainstay of treatment. The fundamental aim of palliative care is to achieve the best quality of life for patients and their carers. This is achieved with specialist symptom control and provision of psychological, social and spiritual (not necessarily religious) support.

Several studies have reported that baseline quality of life is a significant prognostic factor for survival in non-small cell lung cancer (NSCLC) patients[10, 11]. An American un-blinded single centre randomised controlled trial of 151 patients with advanced lung cancer assessed regular early specialist palliative care (SPC) treatment in addition to standard care versus standard care alone, and demonstrated improved HRQoL, fewer symptoms of depression at 12 weeks with reduced utilisation of aggressive endoflife care measures[12]. The same study reported improved survival in the intervention arm, although this was not an *a priori* outcome and the study was not adequately powered to demonstrate such an effect. A more recent study concluded that early SPC might improve HRQoL and satisfaction with care for patients with a large range of solid tumour malignancies[13].

Patients with MPM and advanced lung cancer have very similar symptoms and both conditions have a poor prognosis. However, there are considerable differences in oncology and SPC services between the US and many other countries including the UK, limiting the generalisability of the US study results. The aim of this randomised controlled multicentre trial is to assess if regular early SPC has an effect on HRQoL in patients with newly diagnosed malignant pleural mesothelioma as compared to standard care provided by the UK National Health Service (NHS).

## Methods

### Study objectives

#### Primary aim

The primary aim of our trial is to determine whether regular early SPC in newly diagnosed mesothelioma patients results in improved quality of life 12 weeks after randomisation, as compared to standard care. HRQoL will be measured using the global health status (GHS) subscale of the European Organisation for Research and Treatment of Cancer (EORTC) Quality of Life questionnaire Core 30 (QLQC30), specifically developed to assess the quality of life in cancer patients.

#### Secondary aims

The secondary aims include assessing the impact of regular early SPC on patient HRQoL at 24 weeks, patient mood at 12 and 24 weeks using the General Health Questionnaire-12 (GHQ-12), survival. We will also assess the primary caregiver’s HRQoL (using the ShortForm36 (SF-36) health survey and the family satisfaction with advanced cancer care-2(FAMCARE-2)) and mood (GHQ-12) at 12 and 24 weeks, and additionally at 24 weeks following patient death. Healthcare resource use data and European Quality of Life 5-Dimension questionnaire (EQ-5D) scores will be used to assess cost-effectiveness of the intervention.

#### Exploratory aims

We will explore the significance of the biological and radiological status of the patients at diagnosis on HRQoL outcomes through sub-group analyses.

### Study design and setting

**Figure 1:**
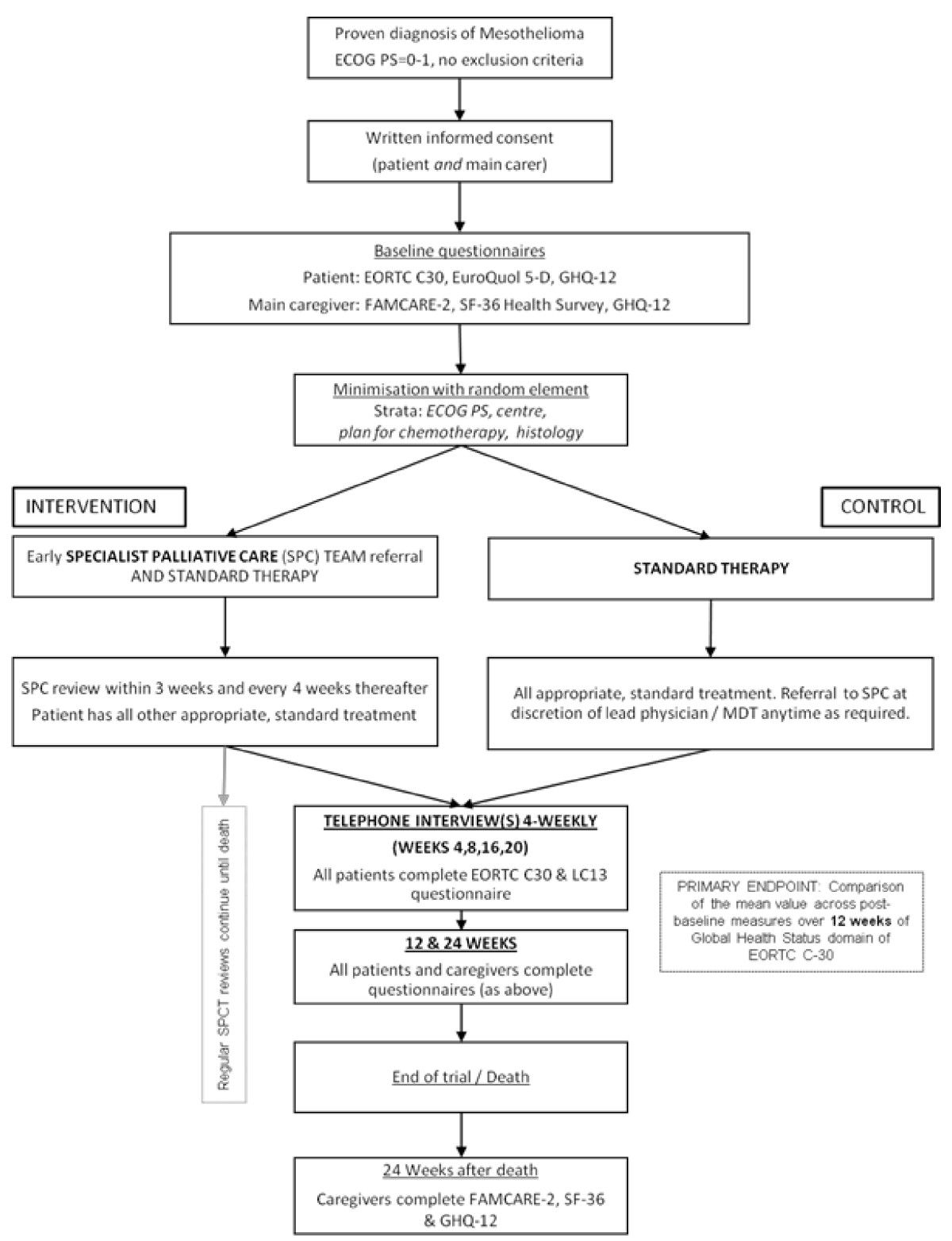
**Study flow diagram.**

This is a multi-centre randomised, non-blinded, parallel group controlled trial comparing early referral to a specialist palliative care team for regular early SPC versus standard care. A summary of the study design is illustrated in Figure 1. Study recruitment commenced in March 2014. It is planned that this trial will be conducted in up to ten centres in England and Scotland.

### Selection of participants

#### Recruitment and informed consent

All new patients discussed at thoracic cancer multidisciplinary team (MDT) meetings will be screened for eligibility and those who meet study criteria will be approached by the study team in conjunction with the Lung Cancer Clinical Nurse Specialists (LCCNS). Suitable patients will be asked to nominate a main carer and both the patient and carer will be provided with verbal and written explanation of the study in the form of an information sheet. If the patient is unable to identify a main carer or the carer declines to participate, the patient may still participate. After adequate time the patient and carer will be invited to a research clinic and informed consent will be obtained by a member of the research team. Informed consent procedures will be carried out in accordance with Good Clinical Practice (GCP).

### Inclusion criteria

1. Histological or cytological confirmation of MPM.
2. European Cooperative Oncology Group (ECOG) Performance Score (PS) of 0 to 1. (asymptomatic patients score 0; symptomatic but fully ambulatory patients score 1).
3. The diagnosis of MPM received within the last six weeks.
4. Ability to provide written informed consent in English and comply with trial procedures.

### Exclusion criteria

1. Other known malignancy within five years (excluding localised squamous cell carcinoma of the skin, cervical intraepithelial neoplasia, grade III and low grade prostate cancer (Gleason score <5, with no metastases)).
2. Significant morbidity which the lead physician or MDT feel will unduly confound or influence HRQoL.
3. Those patients the MDT judge require referral to SPC at the point of diagnosis.
4. Concurrent, or less than three months since participation in another clinical trial that may affect HRQoL.
5. Referral at the time of recruitment for cytoreductive, tumour de-bulking, radical decortication or extrapleural pneumonectomy surgery for MPM. (VideoAssistedThoracoscopicSurgery (VATS) or ‘mini’ thoracotomy for pleurodesis and diagnosis attempts are permissible.)
6. Chemotherapy treatment for MPM initiated prior to consent.
7. A significant history of depression/anxiety/psychiatric illness requiring specialist hospital care within the last twelve months.

### Randomisation and blinding

Following completion of the baseline assessment, eligible patients and their carers will be minimised with a random element in a 1:1 ratio between the intervention and control groups using a centralised randomisation database managed by the Oxford Respiratory Trials Unit (ORTU), Oxford, UK.

Participants will be minimised according to:1) centre, 2) plan for chemotherapy (yes/no), 3) ECOG PS (0 or 1) and 4) histological sub-type (epithelioid versus non-epithelioid (biphasic, sarcomatoid, not defined)).

Due to the nature of the intervention it is not possible to blind participants or the immediate research team to the allocated intervention. Interventions and outcome assessments will therefore be un-blinded. Data analysis will be performed in a blinded fashion.

### Study intervention

Patients randomised to regular early SPC will be seen within three weeks of group allocation by the SPC team. Carers will be encouraged to accompany the patient for these visits. Both the patient and carer will then continue to be seen regularly every four weeks until end of trial (EOT). Consultations will include an assessment of physical, psychological, social and spiritual needs of the participant, with appropriate provision of additional medicine (for example, analgesia) and referral to additional support services as required. By the nature of this care, it is bespoke and further tight definitions or requirements of the intervention are not possible. Similarly, it is difficult to define expected reasons why subjects should choose not to have the intervention, although subjects are free to decline at any point.

To ensure a standard structure and approach to SPC consultations across different centres, the study will use the Sheffield Profile for Assessment and Referral to Care (SPARC) tool[14]and the revised Edmonton Symptom Assessment System (ESAS-r)[15] at initial consultations for intervention group patients and control group patients if and when referred.

### Control arm

The control group will continue to receive all appropriate, routinely provided treatment for MPM currently available within the NHS. This will be initiated by the patient’s general practitioner (GP), the cancer MDT or lead respiratory physician as required. No treatment will be withheld. The referral of patients in the control group to SPC will be at the discretion of the patient’s medical team based on clinical need and according to local timings and practice.

The selection of the comparator as routine care is based on the pragmatic nature of the study, the ethical need to protect vulnerable subjects and to ensure all appropriate care is provided.

### Participant reported outcome measures

Patient and carer HRQoL and mood will be measured using validated pre-specified questionnaires. HRQoL will be assessed using the European Organisation for Research and Treatment of Cancer (EORTC) Quality of Life questionnaire Core 30 (QLQ-C30) and lung cancer supplement (LC13) questionnaires. The EORTC QLQ-C30 has been widely used in clinical trials and been validated in patients with MPM[16]. The EORTC-LC13 adjunct is a further assessment of specific symptoms associated with lung cancer, which are relevant to MPM[16, 17]. The EQ-5D will also be used to measure patient HRQoL and then allow calculation of Quality-Adjusted-Life-Years (QALY). Caregiver HRQoL will be assessed using the 1-week recall SF-36 health survey which measures 8 domains of HRQoL. As it does not target a specific population, age or disease state it is appropriate for measuring family caregivers of patients. The FAMCARE-2 questionnaire has sound psychometric properties and will measure family/carers satisfaction with end-of-life care received by both the patient and their carers. The GHQ-12 is the most widelyused measure for screening for psychiatric morbidity in adults in the UK and will be used to measure patient and carer mood[18].

### Study visit schedule

The patient and carer study visit schedule consists of a combination of face-to-face and telephone contacts as detailed below (see also Table 1).

**Table 1:** **Study visit schedule**

		Baseline	SPC within 3 weeks	4 weeks	8 weeks	12 weeks	16 weeks	20 weeks	24 weeks	Monthly SPC until Death/EOT	24 weeks after death
**Intervention**											
Patient	QLQC-30, LC13, EQ-5D, GHQ-12	X				X			X		
	SPC review		X	X	X	X	X	X	X	X	
	Telephone interview (QLQC-30, LC13)			X	X		X	X			
Carer	GHQ-12, SF-36, FAMCARE-2	X				X			X		X
**Control**											
Patient	QLQC-30, LC13, EQ-5D, GHQ-12	X				X			X		
	Telephone interview (QLQC-30, LC13)			X	X		X	X			
Carer	GHQ-12, SF-36, FAMCARE-2	X				X			X		X

EOT = end of trial;EQ-5D = EuroQol-5D;FAMCARE-2 = family satisfaction with advanced cancer care 2; GHQ-12 = General Health Questionnaire-12; LC13 = lung cancer supplement;,QLQ-C30 = Quality of Life Questionnaire Core 30;SF-36 = Short Form 36;SPC = Specialist Palliative Care.

After consent, the following information will be collected from all patients at the baseline assessment prior to randomisation: patient demographics, date and type of diagnostic pleural procedure, histological subtype of mesothelioma, current performance status (from MDT) and co-morbidities, previous treatments and pleural procedures, treatment plan, medication use, patient HRQoL and mood questionnaires (EORTC-QLQC30, EORTC-LC13, EQ-5D, GHQ-12), neutrophil and lymphocyte values at time of diagnosis, International Mesothelioma Interest Group (IMIG) stage of disease[19] at time of diagnosis (staging data will only be collected from selected sites). From the designated main carer, the following information will be obtained: carer current health utilisation, work status (full or part time, retired), HRQoL and mood questionnaires (SF-36, FAMCARE-2, GHQ-12).

At 12 weeks (primary end point) and 24 weeks, patients and carers from both the intervention and control groups will be seen in clinic and the following information will be documented: patient history including recent treatments and hospital/healthcare utilisation, medication use, patient HRQoL and mood questionnaires (EORTC-QLQC30, EORTC-LC13, EQ-5D, GHQ-12). For carers: healthcare resource utilisation, work status (and days of work missed as appropriate), carer HRQoL and mood questionnaires (SF-36, FAMCARE-2, GHQ-12). Clinic visits will ensure better quality, more complete data for the primary endpoint.

Telephone consultations with patients in both the intervention and control group will be performed at 4, 8, 16 and 20 weeks to obtain the following information: recent treatments and hospital usage, medication use, HRQoL questionnaires (EORTC-QLQC30, EORTC-LC13).

The patient’s vital status will be tracked for the duration of the study. In the event of patient death during the study the following information will be documented: prior hospital/healthcare utilisation and treatments, medication use, date and place of death. After patient death, the carer will be approached by the research team to complete the final questionnaires, 24 weeks after bereavement. Should this process identify psychological morbidity that may require treatment, liaison with the individual’s GP and direct referral to more formal bereavement support services will be considered. The following information will be gathered by telephone at this contact: healthcare utilisation, work status, carer HRQoL and mood questionnaires (SF-36, FAMCARE-2, GHQ-12).

### Safety reporting

Given the nature of mesothelioma, many of the patients involved in the study will have complications from their disease or other treatments (for example, radiotherapy) during the follow-up period, which are unrelated to study participation, and death is also a predicable occurrence during this study. We have still opted to monitor safety during this trial to ensure there are no unexpected consequences of the intervention, and will adhere to the following risk-adapted safety monitoring procedures:

- Recording of all serious adverse events (SAEs) unless judged by the investigator to be part of the patient’s natural disease progression or related to standard treatment
- Any SAE judged by the investigator to be (possibly, probably or definitely) related to the intervention that is also unexpected, should be expedited immediately to the chief investigator and the sponsor, following instructions for expedited reporting within 24 hours of first becoming aware of the event
- Reporting of any observed quality change in events, or any safety concerns judged to be clinically significant and any clinical incident concerning study participants or with an impact on the study

A Data Monitoring Committee was not deemed necessary for this study as the study is not delivering a new intervention and there are no anticipated SAEs or risks from simply changing the timing of this intervention for subjects.

### Statistics

Stata for Windows Release 11(StataCorp LP, College Station, TX, USA) will be used for all the analyses. The study will be reported in accordance with the Consolidated Standards of Reporting Trials statement and ICH Guidelines for GCP. All study data will be managed by the Oxford Respiratory Trials Unit (ORTU) using a bespoke database created using OpenClinica Enterprise Edition software (OpenClinica LLC, Waltham, MA, USA). Confidentiality of participant data will be assured according to GCP.

### Sample size

Assuming a population mean of 55 and a common standard deviation of 22 in GHS/HRQoL for mesothelioma patients[20], a sample size of 78 patients in each arm will be required to detect a 10-point difference in the mean scores between the two groups, with a power of 90% at a 5% two-sided significance level, assuming an autocorrelation of 0.25.

UK National Lung Cancer Audit data demonstrate approximately 6% mortality at 12 weeks for PS 0 to 1 patients - therefore, we will factor a 10% dropout before the primary endpoint is reached at 12 weeks. Therefore, the sample size allowing for a power of 90%, a 5% two-sided significance level, a minimal clinically important difference (MCID) of 10 units in the GHS/HRQoL and a dropout of 10% (n = 16) is 174 patients.

### Clinical outcomes analysis

The primary outcome measure is the transformed global health status subscale score of the QLQ-C30 at 12 weeks from randomisation. Analysis of the primary outcome will be carried out using an analysis of covariance with adjustment for baseline values to compare between groups. Primary and secondary analyses will be carried out on the intention-to-treat (ITT) population.

As a secondary analysis of the primary outcome a mixed-effects regression model will be used, which will account for the repeated measures over time.

Secondary outcomes: for all patient reported secondary outcomes mixed-effects regression models will be used, taking account of the repeated measures. Overall survival from date of randomisation will be analysed using Kaplan-Meir curves and the Log-rank test.

#### Missing data

To minimise total and item non-response, we will perform monthly telephone interviews for quality of life data and clinic appointments for 12- and 24-week questionnaires with the research team. If missing data are substantial (>10%) then multiple imputation techniques will be used for the primary outcome to account for missing data under the missing at random assumption.

### Healthcare economics and utilisation analysis

Resource use items (for example, hospital and hospice bed days, emergency department attendance) will be priced using unit cost schedules such as the NHS Trust financial returns and NHS reference costs[21]. If necessary, finance departments at each of the study centres will be contacted to obtain unit cost information not included in these sources. A within-trial cost-utility analysis will explore the incremental cost per QALY gained by regular early SPC intervention when compared to usual care. QALY will be generated by combining utility information obtained from the EQ-5D and survival data, all collected as part of the trial. Resource use, cost and effect results will be reported as means with standard deviations, with mean differences between the two patient groups reported with 95% confidence intervals. Incremental cost-effectiveness will be calculated by dividing the difference in costs by the difference in effects. Uncertainty around the incremental cost-effectiveness ratio will be explored using non-parametric bootstrapping[22].

### Ethics

A favourable ethical approval for this study has been granted by the National Research Ethics Service (NRES) Committee, London (Hampstead), reference 12/LO/0078. The trial will be conducted according to the Declaration of Helsinki[23].

The study intervention is regular early specialist symptom control treatment which will be carried out by SPC teams and is considered to have no additional safety risk compared with usual clinical practice. Despite being an interventional design, the process involved is not novel and is the usual standard of care for these patients, albeit normally provided at a much later stage in the natural history of this illness. This study will simply provide the same care, delivered at an earlier time point.

The key ethical issues are considered to be as follows: no treatments or care will be withheld at any point in the study, with all subjects (in either arm) able to receive any care considered appropriate by the treating physicians. The additional regular SPC input will continue beyond the EOT until death, with remaining subjects integrated into existing palliative care services.

### Funding source, sponsor and trial oversight

This study has been funded through a competitive grant application to the British Lung Foundation (BLF). The sponsor for this trial is Portsmouth Hospitals NHS Trust. In collaboration with the sponsor, the Oxford Respiratory Trials Unit (ORTU) will oversee quality assurance and trial conduct with routine and for-cause audit performed in accordance with GCP guidelines as appropriate.

### Dissemination policy

The full trial results will be published in a high impact medical journal; in addition, lay summaries will be developed with public patient involvement members and disseminated through respiratory and cancer charities and support groups. Lay and scientific summaries will be placed on study website http://www.respect-meso.org.

## Discussion

This study will inform clinicians of a potentially novel way to care for patients with this high symptom-burden disease for which there is no cure, and limited treatment options. The additional aspect of healthcare resource utilisation analysis will allow further interpretation of the results and may inform clinicians and policy-makers as to a more complete understanding as to the implications of this intervention.

In the design of the RESPECT-MESO study we have attempted to allow as pragmatic an approach as possible, to ensure as wide as possible external applicability of the results, within the confines of ensuring internal validity and appropriate scientific rigor. One of the central difficult decisions made was to only include subjects with an ECOG PS of 0 to 1. This was based on UK National Lung Cancer Audit data demonstrating approximately 24% mortality at 12 weeks for PS 2 patients with mesothelioma. Such attrition would have significantly increased the challenge of obtaining adequate data for the primary endpoint at 12 weeks. Nevertheless, UK data demonstrate that 70 to 80% of patients with mesothelioma have a PS of 0 to 1 at the time of diagnosis[24–27], so our approach will allow the inclusion of the majority of cases at presentation.

One of the additional key challenges encountered in the setup of the study was developing the necessary links with SPC services. This has been addressed by early engagement with SPC teams and offering SPC team leads the opportunity of taking on local co-principal investigator roles. When screening potential host sites for suitability we have used a feasibility assessment focusing on the availability of SPC services and asking sites to nominate both respiratory and SPC leads for the study.

### Trial status

The study is now open to recruitment at Queen Alexandra Hospital, Portsmouth, the lead centre for the RESPECT-Meso study. Further centres will be opening for recruitment throughout the United Kingdom over 2014/15. Further information can be found at http://www.respect-meso.org.

## Authors’ original submitted files for images

Below are the links to the authors’ original submitted files for images. Authors’ original file for figure 1

## Abbreviations

ANCOVA: analysis of covariance
BLF: British Lung Foundation
ECOG: European Cooperative Oncology Group
EORTC: European Organisation for Research and Treatment of Cancer
EOT: end of trial
EQ-5D: European Quality of Life 5-Dimension questionnaire
ESAS-r: Revised Edmonton Symptom Assessment System
FAMCARE-2: FAMily satisfaction with advanced cancer CARE-2
GCP: Good Clinical Practice
GHQ-12: General Health Questionnaire-12
GHS: global health status
GP: general practitioner
HRQoL: Health Related Quality of Life
ICH: International Conference of Harmonisation
IMIG: International Mesothelioma Interest Group
ITT: intention-to-treat
LC13: lung cancer supplement
LCCNS: lung cancer clinical nurse specialists
MCID: minimal clinically important difference
MDT: multidisciplinary team
MPM: malignant pleural mesothelioma
NHS: National Health Service
NRES: National Research Ethics Service
NSCLC: non-small cell lung cancer
ORTU: Oxford Respiratory Trials Unit
PS: performance score
QALY: Quality-Adjusted-Life-Years
QLQ-C30: Quality of Life questionnaire Core 30
SAE: serious adverse event
SF-36: Short Form 36
SPARC: Sheffield Profile for Assessment and Referral to Care
SPC: specialist palliative care
UK: United Kingdom
US: United States.
