# Supplementary material for: A multicentre non-blinded randomised controlled trial to assess the impact of regular early specialist symptom control treatment on quality of life in malignant mesothelioma (RESPECT-MESO): study protocol for a randomised controlled trial
Source: Trials. 2014 Sep 19;15:367. doi: 10.1186/1745-6215-15-367 (PMC4180732; doi:10.1186/1745-6215-15-367)
Supplement: Supplementary file 1 — Authors’ original file for figure 1 [file 13063_2014_2238_MOESM1_ESM.pdf]

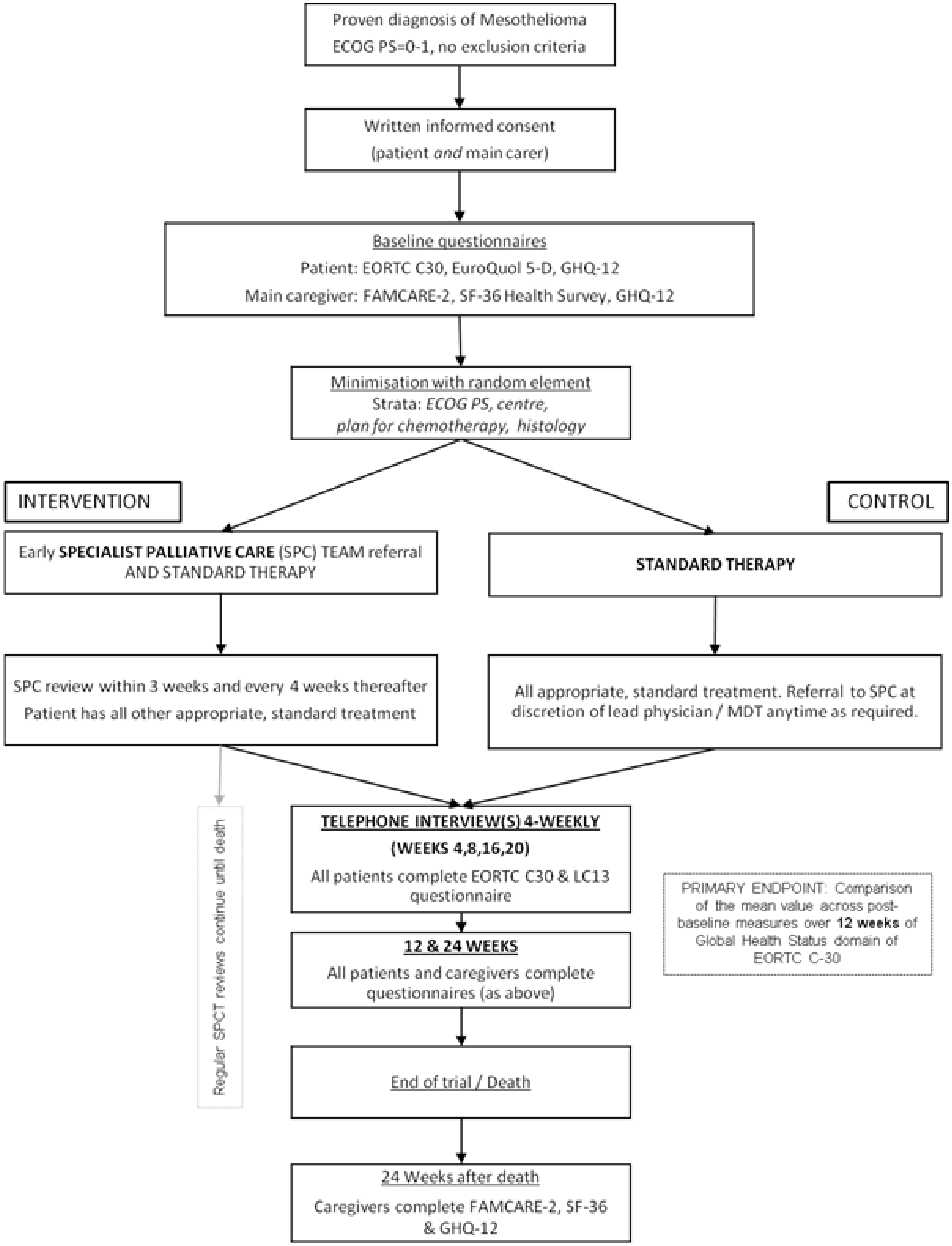
Proven diagnosis of Mesothelioma
ECOG PS=0-1, no exclusion criteria
Written informed consent
(patient and main carer)
Baseline questionnaires
Patient: EORTC C30, EuroQuol 5-D, GHQ-12
Main caregiver: FAMCARE-2, SF-36 Health Survey, GHQ-12
Minimisation with random element
Strata: ECOG PS, centre, plan for chemotherapy, histology
INTERVENTION
CONTROL
Early SPECIALIST PALLIATIVE CARE (SPC) TEAM referral AND STANDARD THERAPY
STANDARD THERAPY
SPC review within 3 weeks and every 4 weeks thereafter
Patient has all other appropriate, standard treatment
All appropriate, standard treatment. Referral to SPC at discretion of lead physician / MDT anytime as required.
Regular SPCT reviews continue until death
TELEPHONE INTERVIEW(S) 4-WEEKLY
(WEEKS 4,8,16,20)
All patients complete EORTC C30 & LC13 questionnaire
PRIMARY ENDPOINT: Comparison of the mean value across post-baseline measures over 12 weeks of Global Health Status domain of EORTC C-30
12 & 24 WEEKS
All patients and caregivers complete questionnaires (as above)
End of trial / Death
24 Weeks after death
Caregivers complete FAMCARE-2, SF-36 & GHQ-12
